# Hemispheric differences in the surgical outcomes of patients with traumatic acute subdural hematoma

**DOI:** 10.1186/1477-5751-13-10

**Published:** 2014-05-31

**Authors:** Joji Inamasu, Mitsuhiro Hasegawa, Takuro Hayashi, Yoko Kato, Yuichi Hirose

**Affiliations:** 1 Department of Neurosurgery, Fujita Health University Hospital, 1-98 Kutsukake, Toyoake 470-1192, Japan

**Keywords:** Acute subdural hematoma, Hemispheric differences, Outcomes, Surgery

## Abstract

**Background:**

Our assumption that prognosis of patients with traumatic acute subdural hematoma (ASDH) does not differ significantly according to the hemispheric laterality has never been verified.

**Methods:**

A review of the charts/radiographic images of 61 adult traumatic ASDH patients (33 left/28 right) was conducted. Intergroup comparison was made on the demographics, autonomic/laboratory data, and outcomes (90-day mortality rate). Based on the presence of concomitant brain contusion, patients were further quadrichotomized as: left ASDH with contusion (n = 14), right ASDH with contusion (n = 16), left ASDH without contusion (n = 19), and right ASDH without contusion (n = 12). Comparisons were made on demographic and outcome variables between the left ASDH with contusion and right ASDH with contusion, and between the left ASDH without contusion and right ASDH without contusion. Multivariate regression analysis was conducted to identify clinical variables correlated with fatality.

**Results:**

There were no significant differences in the demographic, autonomic, and laboratory data between the left and right ASDH patients. However, 90-day mortality rate was significantly higher in the left ASDH patients when concomitant contusion was present (79% vs. 25%, *p* = 0.009). However, there were no significant hemispheric differences in the mortality rate among those without contusion (32% vs. 33%, *p* = 0.77). Multivariate regression analysis showed that left ASDH was correlated with fatality among those with contusion (OR: 6.620; 95% CI: 1.219-46.249).

**Conclusions:**

This study is probably the first to report that the left ASDH patients fared substantially worse than the right-sided counterparts. Future trials on traumatic ASDHs may benefit from considering hemispheric differences in the outcomes.

## Background

Traumatic acute subdural hematoma (ASDH) with marked brain shift is a life-threatening condition for which prompt evacuation of hematoma is mandatory. Several clinical factors predictive of surgical outcomes have been identified, including patient age and preoperative Glasgow Coma Scale (GCS) scores [1,2]. However, whether hemispheric laterality (i.e., left vs. right) of the hematoma affects the outcomes has never been evaluated in the past. Substantial hemispheric differences in mortality rate and autonomic parameters have been reported in patients with supratentorial stroke [3-11], and potentially, such differences may also exist in patients with traumatic ASDH. This study was conducted to explore the possibility of hemispheric differences in the surgical outcomes of adult traumatic ASDH patients.

## Patients and methods

### Study population

This is a single-center retrospective study. The study protocol was approved by our institutional internal review board. Between January 2005 and June 2012, a total of 78 adult patients (>15 years of age) with symptomatic traumatic ASDH were admitted to our institution and underwent surgery within 24 h of injury. Among them, ASDHs in 9 patients were not related to trauma in etiology and were excluded from analysis. Another patient, who sustained bilateral traumatic ASDH and underwent bilateral craniotomies, was also excluded. Seven ASDH patients who sustained multiple systemic injuries were also excluded. Charts and radiographic images of the remaining 61 patients with traumatic ASDH secondary to isolated closed head injury were thoroughly reviewed. A blinded board-certified neurosurgeon determined whether a concomitant brain contusion was present or not by reviewing their preoperative computed tomography (CT) scans. The volume of contusional hematoma was estimated using the ABC/2 method [12], and when there were multiple contusions in a single patient, a total sum of the hematoma volume was described. The same neurosurgeon also conducted CT measurement of length of the midline shift. Initially, the 61 patients were dichotomized on the basis of hematoma laterality (i.e., left vs. right), and as a result, 33 left ASDH and 28 right ASDH patients were identified. Demographic, autonomic/laboratory data, and outcomes were compared between the two groups. The 61 patients were further quadrichotomized on the basis of presence of concomitant contusion: left ASDH with contusion (n = 14), right ASDH with contusion (n = 16), left ASDH without contusion (n = 19), and right ASDH without contusion (n = 12). Subsequently, demographic and outcome data were compared within the same categories.

### Clinical management

Shortly after CT diagnosis of traumatic ASDH was established, patients were admitted to the neurointensive care unit of our institution. Surgery was indicated on the basis of the Japanese Guidelines for the Management of Severe Head Injury [13]. Patients underwent either craniotomy or decompressive craniectomy for hematoma removal, and decision of which surgery to perform was made intraoperatively by an attending neurosurgeon on-call. In patients with concomitant contusion, internal decompression to remove a contusional hematoma was not performed routinely and the decision to perform it was also at the discretion of the attending neurosurgeon. Preoperative consciousness levels were evaluated with the Glasgow Coma Scale (GCS) scores. Intravenous mannitol was used routinely perioperatively. However, placement of intracranial pressure (ICP) sensors was not routine.

### Autonomic parameters and laboratory data

Systolic blood pressures (SBPs)/diastolic blood pressures (DBPs) and heart rates (HRs) were compared between the 33 left ASDH and 28 right ASDH patients. After emergency department (ED) arrival, noninvasive BPs were measured repeatedly with an automated sphygmomanometer on 5–10 min intervals. The highest SBPs and DBPs were compared between the two groups. Complete blood count, blood chemistry, coagulation profiles and arterial blood gas values, all of which were routinely obtained in our ED, were also compared. All blood samples were collected within 1 h of ED arrival.

### Outcomes

Ninety-day mortality rate, frequency of intra- or postoperative brain swelling refractory to treatment, and mean hospital stay were compared between the 33 left ASDH and 28 right ASDH patients. Furthermore, subgroup analysis was conducted on these variables after quadrichotomization of the 61 patients based on the presence of contusion (14 left ASDH with contusion vs. 16 right ASDH with contusion, and 19 left ASDH without contusion vs. 12 right ASDH without contusion). The volume and location of contusional hematoma was also compared within the former two subgroups. For survivors, activity of daily living 90 days after surgery was assessed with modified Rankin scale (mRS). Furthermore, multivariate regression analysis was conducted among patients with contusion to identify clinical factors correlated with fatal outcomes.

### Statistical analysis

For comparison of categorical and continuous variables, Chi-square test and unpaired *t*-test was used, respectively. JMP Clinical (SAS, Cary, NC, USA) was used for statistical analyses. Data were shown by mean ± SD, and *p* < 0.05 was deemed statistically significant.

## Results

### Demographics

Demographic variables compared between the 33 left ASDH and 28 right ASDH patients included age, gender ratio, preoperative GCS scores, midline shift length on preoperative CT, ratio of high- vs. low-energy trauma, preoperative use of anticoagulants/antiplatelets, history of hypertension, and diabetes. There were no significant intergroup differences in any of the variables described above (Table 1).

**Table 1:** Hemispheric differences in the demographics of patients with traumatic acute subdural hematoma treated surgically

	**Left ASDH**	**Right ASDH**	***p*-value**
	**(n = 33)**	**(n = 28)**	
Age (y)	65.2 ± 17.0	65.5 ± 18.2	0.96
Male: Female	22:11	23:5	0.28
Preoperative GCS score	7.3 ± 4.3	7.0 ± 3.8	0.82
Cause of traumatic ASDH	MVA 9, fall from height 3, ground-level fall 21	MVA 12, fall from height 3, ground-level fall 13	N/A
High-energy vs. Low-energy trauma	12:21	15:13	0.18
ASDH w/contusion vs. ASDH w/o contusion	14:19	16:12	0.25
Midline shift on CT scan (mm)	13.3 ± 5.9	12.0 ± 5.3	0.37
Anticoagulant/Antiplatelet use	10 (30%)	5 (18%)	0.41
Hypertension	10 (30%)	13 (46%)	0.20
Diabetics	3 (9%)	5 (18%)	0.27

ASDH: acute subdural hematoma; CT: computed tomography; GCS: Glasgow Coma Scale; MVA: motor vehicle accident; N/A: not applicable.

### Autonomic parameters and laboratory data

Mean admission SBPs were 172.7 ± 34.2 mmHg in left ASDH and 184.0 ± 38.1 mmHg in right ASDH patients. Although the right ASDH patients exhibited higher SBPs, The difference was not statistically significant (*p* = 0.20) (Table 2). Mean admission DBPs were 89.6 ± 17.2 mmHg in left ASDH and 95.7 ± 20.7 mmHg in right ASDH patients. The difference was not statistically significant (*p* = 0.21). Mean admission HRs (beats/min) were 86.6 ± 21.6 in left ASDH and 88.5 ± 25.9 in right ASDH patients. The difference was not statistically significant (*p* = 0.74). Laboratory data compared were: blood hemoglobin levels, platelet counts, prothrombin time/international normalized ratio, d-dimer levels, blood glucose levels, and arterial glucose levels. There were no significant intergroup differences in any of the variables described above (Table 2).

**Table 2:** Hemispheric differences in the autonomic/laboratory data of patients with acute subdural hematoma treated surgically

	**Left ASDH**	**Right ASDH**	***p*-value**
	**(n = 33)**	**(n = 28)**	
Systolic blood pressures (mmHg)	172.7 ± 34.2	184.0 ± 38.1	0.20
Diastolic blood pressures (mmHg)	89.6 ± 17.2	95.7 ± 20.7	0.21
Heart rates (beats per min)	86.6 ± 21.6	88.5 ± 25.9	0.74
Hemoglobin (g/dL)	12.4 ± 2.2	12.7 ± 3.2	0.63
Platelet count (×10^4^/μL)	16.3 ± 6.0	18.6 ± 10.6	0.29
PT-INR	1.18 ± 0.30	1.12 ± 0.20	0.44
D-dimer (μg/mL)	38.9 ± 54.9	61.1 ± 79.5	0.42
Blood glucose (mg/dL)	184.7 ± 83.8	198.1 ± 86.2	0.54
Arterial lactate (mg/dL)	24.2 ± 18.5	33.1 ± 22.8	0.31

ASDH: acute subdural hematoma; PT-INR: prothrombin time-international normalization ratio.

### Outcomes

Outcome variables compared between the 33 left ASDH and 28 right ASDH patients included ratio of craniotomy vs. decompressive craniectomy, frequency of intractable brain swelling during or after surgery, 90-day mortality rate, and mean hospital stay. There was no significant intergroup difference in the ratio of craniotomy vs. decompressive craniectomy (*p* = 0.22) (Table 3). However, frequency of intractable brain swelling (45% vs. 21%, *p* = 0.09) and 90-day mortality rate (52% vs. 29%, *p* = 0.12) trended to be higher in the left ASDH patients (Table 3). Mean hospital stays were significantly longer for right ASDH patients (18.4 ± 15.2 days vs. 37.9 ± 33.6 days, *p* = 0.005).

**Table 3:** Hemispheric differences in outcomes of patients with traumatic acute subdural hematoma treated surgically

	**Left ASDH**	**Right ASDH**	***p*-value**
	**(n = 33)**	**(n = 28)**	
Craniotomy: Craniectomy	10:23	4:24	0.22
Intractable brain swelling	15 (45%)	6 (21%)	0.09
90-day mortality	17 (52%)	8 (29%)	0.12
Mean hospital stay (d)	18.4 ± 15.2	37.9 ± 33.6	0.005*

ASDH: acute subdural hematoma.

*Statistically significant.

Subsequently, demographic and outcome variables were compared between 14 left ASDH patients with contusion and 16 right ASDH patients with contusion, and also between 19 left ASDH patients without contusion and 12 right ASDH patients without contusion. The left ASDH patients with contusion sustained significantly higher frequency of intractable brain swelling (71% vs. 25%, *p* = 0.03) and 90-day mortality rate (79% vs. 25%, *p* = 0.009) compared with the right-sided counterparts (Table 4). There were no significant differences in the total hematoma volume (14.7 ± 9.2 mL vs. 19.6 ± 21.1 mL, *p* = 0.43) or frequency of patients who underwent removal of the contusional hematoma (14% vs. 19%, p = 1.00). The frontal lobes were the most frequent sites for the concomitant contusion (12 in left ASDH vs. 13 in right ASDH). By contrast, involvement of the temporal lobes was less frequent (3 in left ASDH vs. 4 in right ASDH). In patients without contusion, there were no significant intergroup differences in the frequency of intractable brain swelling (26% vs. 17%, *p* = 0.68) or 90-day mortality rate (32% vs. 33%, *p* = 0.77) (Table 4).

**Table 4:** Outcomes of acute subdural hematoma patients quadrichotomized on the basis of presence of concomitant contusion

	**Left ASDH w/ contusion**	**Right ASDH w/ contusion**	***p*-value**	**Left ASDH w/ contusion**	**Right ASDH w/ contusion**	***p*-value**
	**(n = 14)**	**(n = 16)**		**(n = 19)**	**(n = 12)**	
Age	69.2 ± 12.9	59.7 ± 19.9	0.16	63.0 ± 19.4	70.7 ± 14.5	0.20
Preoperative GCS score	6.8 ± 3.3	5.8 ± 2.7	0.40	7.6 ± 4.8	8.4 ± 4.6	0.32
Volume of contusional ICH (mL)	14.7 ± 9.2	19.6 ± 21.0	0.43	N/A	N/A	N/A
Locations of contusional ICH^†^	F12, T3	F13, T4, others 2	N/A	N/A	N/A	N/A
Concomitant ICH removal	2 (14%)	3 (19%)	1.00	N/A	N/A	N/A
Craniotomy: Craniectomy	1:13	0:16	1.00	10:9	7:5	0.95
Intractable brain swelling	10 (71%)	4 (25%)	0.03*	5 (26%)	2 (17%)	0.68
90-day mortality	11 (79%)	4 (25%)	0.009**	6 (32%)	4 (33%)	0.77

*,**Statistically significant.

^†^1 patient in left ASDH group and 2 patients in right ASDH group had multiple lobar contusions.

ASDH: acute subdural hematoma; ICH: intracerebral hemorrhage; F: frontal lobe; GCS: Glasgow Coma Scale; N/A: not applicable; w/: with; w/o: without.

Multivariate regression analysis was performed with variables including age, gender, laterality of ASDH, admission GCS scores ≤ 8, and estimated total hematoma volume [14]. Left-sided ASDH was correlated with fatal outcomes 90 day after surgery (OR, 6.620; 95% CI: 1.219-46.249, *p* = 0.03). By contrast, none of the other variables were correlated with fatal outcomes. The results were summarized in Table 5.

**Table 5:** Multivariate regression analysis to identify variables correlated with fatality in patients with concomitant contusion

**Clinical variables**	**OR**	**95% CI**	***p***
Age	1.072	0.979-1.174	0.13
Female sex	0.457	0.049-4.268	0.49
Left-sided ASDH	6.620	1.219-46.249	0.03*
Admission GCS score ≤ 8	0.966	0.097-9.640	0.98
Total hematoma volume	0.995	0.934-1.059	0.87

ASDH: acute subdural hematoma; CI: confidence interval; GCS: Glasgow Coma Scale; OR: odds ratio.

*Statistically significant.

The causes of death (17 left ASDH vs. 8 right ASDH) were summarized in Table 6. In both groups, the great majority of patients died from intractable brain swelling. A few patients in each group died from non-cerebral causes. For the 36 survivors (16 left ASDH vs. 20 right ASDH), mean 90-day mRS scores were 3.5 ± 1.0 in left ASDH and 4.0 ± 1.1 in right ASDH patients. Although the left-sided survivors faired slightly better, the difference was not statistically significant (*p* = 0.17). The mean hospital stays were 33.6 ± 26.5 days for left ASDH and 42.3 ± 32.5 days for right ASDH survivors. The difference was not statistically significant (*p* = 0.42).

**Table 6:** Hemispheric differences in the causes of 25 deceased patients and 90-day outcomes in 36 surviving patients with acute subdural hematoma

	**Deceased left ASDH**	**Deceased right ASDH**	**Surviving left ASDH**	**Surviving right ASDH**	***p*-value**
	**(n = 17)**	**(n = 8)**	**(n = 16)**	**(n = 20)**	
Intractable brain swelling	15 (88%)	6 (75%)	N/A	N/A	
Infection	1 (6%)	1 (12.5%)	N/A	N/A	
Adverse cardiac event	1 (6%)	1 (12.5%)	N/A	N/A	
Modified Rankin Scale	6 (by definition)	6 (by definition)	Median: 3	Median: 4	0.17
			Mean: 3.5 ± 1.0	Mean: 4.0. ± 1.1	
Mean hospital stay for survivors (days)	N/A	N/A	33.6 ± 26.5	42.3 ± 32.5	0.42

### Case illustrations

**Figure 1:**
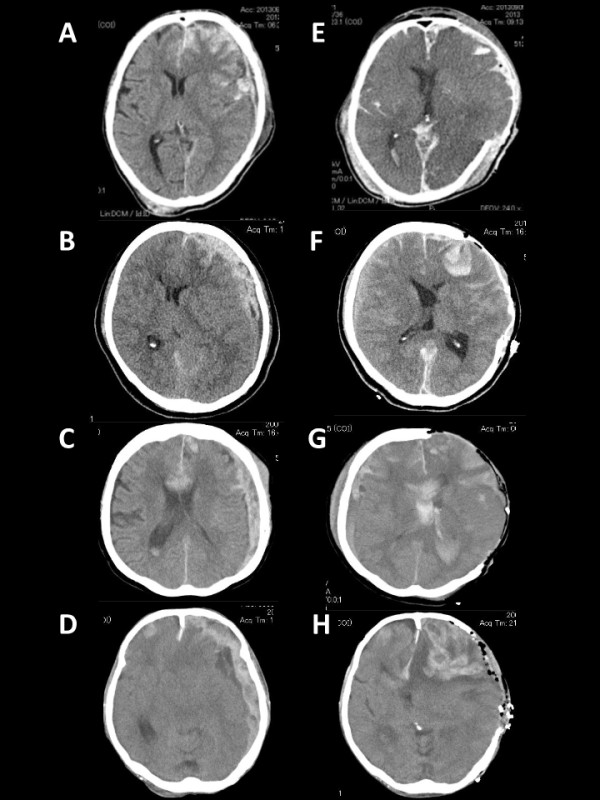
**Pre- (A, B, C, D) and postoperative (E, F, G, H) computed tomography (CT) scans of four left acute traumatic subdural hematoma (ASDH) patients with contusion, all of whom sustained fatal brain swelling. A**, **E**: a 64-year-old man with Glasgow Coma Scale (GCS) score of 3; **B**, **F**: a 73-year-old woman with GCS score of 6; **C**, **G**: a 71-year-old woman with GCS score of 11; **D**, **H**: a 68-year-old man with GCS score of 4. All postoperative CT scans were obtained within 24 h of surgery.

**Figure 2:**
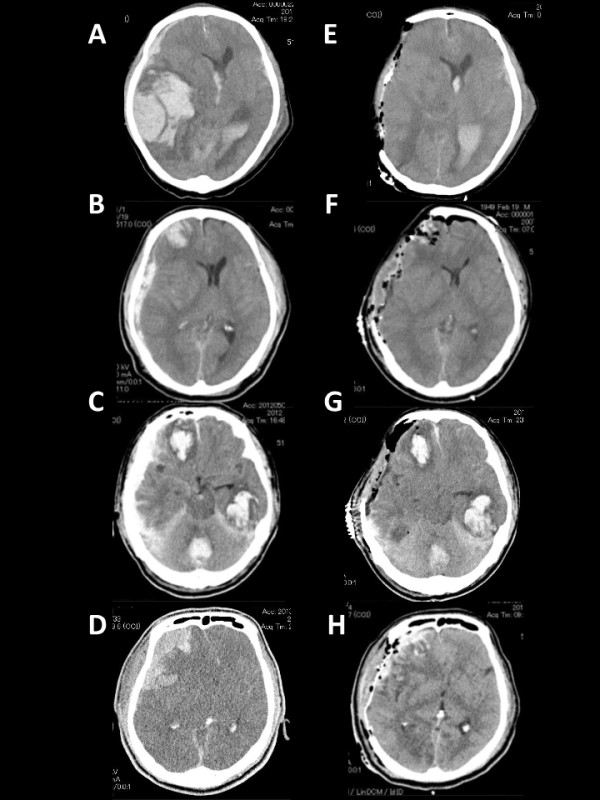
**Pre- (A, B, C, D) and postoperative (E, F, G, H) CT scans of four right ASDH patients with contusion, none of whom sustained intractable brain swelling. A**, **E**: a 57-year-old man with GCS score of 5; **B**, **F**: a 58-year-old man with GCS score of 3; **C**, **G**: a 79-year-old man with GCS score of 5; **D**, **H**: a 64-year-old man with GCS score of 6. All postoperative CT scans were obtained within 24 h of surgery.

Pre- and postoperative CT scans of four patients with left ASDH with contusion (Figure 1A-H), all of whom sustained intractable brain swelling and died, and other four patients with right ASDH with contusion (Figure 2A-H), none of whom sustained intractable brain swelling and died, were shown to highlight possible hemispheric differences in the degree of severe brain swelling. All postoperative CT scans were obtained within 24 h of surgery.

## Discussion

Hemispheric differences in the short- and long-term outcomes of ischemic stroke patients have been known for several decades and studied extensively [3-11]. Patients with the right insular stroke showed significantly higher 1- to 2-year mortality rates compared with the left-sided counterparts [6-9]. On the other hand, patients with the left hemispheric stroke were shown to sustain significantly higher in-hospital mortality rate compared with the right-sided counterparts [10]. However, whether the laterality of hematoma may affect the surgical outcomes of traumatic ASDH patients has never been evaluated in the past: almost all of the earlier studies on supratentorial traumatic ASDH have been based on the assumption that surgical outcomes are equal regardless of the laterality of hematoma, and therefore, this study may probably be the first to report that the left ASDH patients fared substantially worse compared with the right-sided counterparts. Most deaths in both groups were attributable to intractable brain swelling during or after surgery (Table 6). Probably because of their higher survival rate, hospital stay was significantly longer in the right ASDH patients (Table 4). Interestingly, there was no significant hemispheric difference in the mean hospital stay among survivors (Table 6). The hemispheric difference in the outcomes was significant only when concomitant contusion was present: there was no significant difference in the mortality rate or frequency of intractable brain swelling in ASDH patients without contusion (Table 4). Left-sided ASDH with contusion as a predictor of poor outcomes was also shown by multivariate regression analysis (Table 5). That the left ASDH patients with contusion fared particularly poorly can be useful information to neurosurgeons/emergency physicians who take initial care of many traumatic ASDH patients.

In ischemic stroke patients, the hemispheric difference in the outcomes has mainly been attributed to the difference in autonomic dysfunctions following the left vs. right insula [3-11]: the insula, a part of the medial temporal lobe, is pivotal in the integration of various autonomic inputs [3-11]. Ischemic injury to the left insula resulted in more profound cardiac dysfunction, lower plasma catecholamine levels and subsequently, lower blood pressures compared with the right insular injury [15-18]. The left ASDH patients *did* exhibit lower blood pressures than the right ASDH patients in this study, although the difference was not significant (Table 2). Studies on hemispheric ischemic stroke have suggested that the left cerebral hemisphere may have greater metabolic demands than the right side under ischemic condition [10,19], and potentially, the same phenomenon may also have occurred in ASDH patients. ICP is elevated more frequently and profoundly in patients with concomitant contusion [1,2], and left ASDH patients with contusion may have experienced intractable brain swelling more frequently because of combination of cardiac depression, high metabolic demand and elevated ICP. The volume of contusional hematoma by itself may not be predictive of fatal outcomes (Table 5), and there were no significant hemispheric differences in the total hematoma volume (Table 4).

It remains to be seen whether the hemispheric differences in the outcomes of traumatic ASDH patients could totally be attributable to the left-right difference in insular injury, since ASDH differs from ischemic stroke in that a lesion exists outside of the brain parenchyma in the former: only a handful of ASDH patients in our cohort sustained direct injury to the temporal lobes (Table 4), and furthermore, there is insufficient evidence to prove our speculation that temporal lobe compression by severe ASDH result in temporary insular injury or dysfunction. Studies that evaluated impairment of cerebral autoregulation in traumatic brain injury patients found significant asymmetry of the autoregulatory index between the injured and intact hemisphere [20,21]. However, these studies failed to show that the left ASDH patients were more prone to develop autoregulatory impairment compared with the right-sided counterparts [20,21].

There are not a few limitations to this study. First, this is a retrospective study. Because of emergency setting and limitation in time, potentially useful parameters to assess hemispheric lateralization in autonomic functions such as heart rate variability, frequently used in stroke patients [22,23], could not be evaluated. Second, information pertaining to the handedness of each patient was difficult to obtain and was not evaluated. Third, ICP had not been measured routinely in our cohort and it remains unclear whether there might have been significant hemispheric differences in the ICP values of traumatic ASDH patients with brain contusion. Finally, it should be noted that the right-sided ASDH patients did develop intractable brain swelling, although no such cases were depicted in the Case illustration section. Despite the aforementioned limitations, we expect that this study will lead to the further elucidation of mechanisms involved in the hemispheric differences in the autonomic parameters and outcomes of traumatic ASDH patients. It is obvious that our findings need to be scrutinized by other groups for reproducibility; however, the revalidation process may not be difficult considering the fact that ASDH is a relatively common brain injury, and most institutions may have their own databases [1,2,24].

## Conclusions

This study may be the first to report that the left ASDH patients fared significantly worse compared with the right-sided counterparts particularly when concomitant brain contusion was present, and may serve as useful prognostic information on traumatic ASDH patients for neurosurgeons. Future trials on traumatic ASDHs may benefit from considering potential hemispheric differences in the outcomes and other demographic variables.

## Competing interests

On behalf of all authors, the corresponding author states that there is no financial o other conflict of interests.

## Authors’ contributions

JI: data acquisition, analysis, interpretation, and drafting of manuscript. MH: data acquisition and interpretation. TH: data acquisition and interpretation. YK: data interpretation and supervision of statistical analysis. YH: Study conception and supervision of statistical analysis. All authors read and approved the final manuscript.
